# Neuropeptides as Targets for the Development of Anticonvulsant Drugs

**DOI:** 10.1007/s12035-014-8669-x

**Published:** 2014-04-06

**Authors:** Elke Clynen, Ann Swijsen, Marjolein Raijmakers, Govert Hoogland, Jean-Michel Rigo

**Affiliations:** 1 Biomedical Research Institute BIOMED, Hasselt University, Martelarenlaan 42, 3500 Hasselt, Belgium; 2 Department of Neurosurgery, School of Mental Health and Neurosciences, University Medical Center Maastricht, P.O. Box 616, 6200 MD Maastricht, The Netherlands

**Keywords:** Animal seizure models, Antiepileptic drugs, Epilepsy, Febrile seizures, Neuropeptides, Seizures

## Abstract

Epilepsy is a common neurological disorder characterized by recurrent seizures. These seizures are due to abnormal excessive and synchronous neuronal activity in the brain caused by a disruption of the delicate balance between excitation and inhibition. Neuropeptides can contribute to such misbalance by modulating the effect of classical excitatory and inhibitory neurotransmitters. In this review, we discuss 21 different neuropeptides that have been linked to seizure disorders. These neuropeptides show an aberrant expression and/or release in animal seizure models and/or epilepsy patients. Many of these endogenous peptides, like adrenocorticotropic hormone, angiotensin, cholecystokinin, cortistatin, dynorphin, galanin, ghrelin, neuropeptide Y, neurotensin, somatostatin, and thyrotropin-releasing hormone, are able to suppress seizures in the brain. Other neuropeptides, such as arginine-vasopressine peptide, corticotropin-releasing hormone, enkephalin, β-endorphin, pituitary adenylate cyclase-activating polypeptide, and tachykinins have proconvulsive properties. For oxytocin and melanin-concentrating hormone both pro- and anticonvulsive effects have been reported, and this seems to be dose or time dependent. All these neuropeptides and their receptors are interesting targets for the development of new antiepileptic drugs. Other neuropeptides such as nesfatin-1 and vasoactive intestinal peptide have been less studied in this field; however, as nesfatin-1 levels change over the course of epilepsy, this can be considered as an interesting marker to diagnose patients who have suffered a recent epileptic seizure.

## Introduction

Epilepsy is one of the most common neurological disorders, affecting about 70 million people worldwide [1]. In some cases, epilepsy has a genetic etiology, while in other cases precipitating events such as head trauma, inflammation, stroke, tumors, or prolonged febrile seizures during childhood lead to the development of epilepsy. The fundamental characteristic of epilepsy is the occurrence of recurrent, unprovoked seizures. These seizures are a result of excessive electrical discharges in a group of neurons that fire with a synchrony that never occurs during normal behavior. They are caused by a disruption of the delicate balance between inhibition and excitation in the brain. On the neurotransmitter level, two major players contribute to such misbalance: the inhibitory transmitter GABA and the excitatory transmitter glutamate. Neuropeptides are powerful modulators of these classical neurotransmitters, either by modifying their release or by regulating their effects at the receptor level, and can hence influence the balance between inhibition and excitation. Neuropeptides also modulate monoaminergic transmission, such as dopamine and serotonin, and can thereby also alter brain excitability. Furthermore, as they are stored in large dense core granules, neuropeptides are likely released during sustained high-frequency activity (5–40 Hz), such as that occurring during epileptiform seizures.

In the past years, numerous neuropeptides have been linked to epilepsy [2, 3]. Aberrant neuropeptide levels were found in plasma, cerebrospinal fluid (CSF) and resected tissues from epilepsy patients and in different animal seizure models (Table 1).^1^ Some of these neuropeptides have been implicated in the regulation of seizure susceptibility. In fact, many endogenous neuropeptides display anticonvulsive effects in animal models of epilepsy. Hence, these neuropeptides and their receptors are attractive targets for the development of new antiepileptic drugs (AEDs). Other neuropeptides show proconvulsive effects, which makes their receptors also interesting targets for suppressing seizures using, e.g., receptor-specific antagonists. AEDs suppress seizure activity and thereby greatly improve the quality of life of epilepsy patients. Unfortunately, at present, only two thirds of patients achieve good seizure control under pharmacological treatment [1]. Therefore, new drug targets are needed.

**Table 1:** Overview of animal models referred to in this review [216]

Chemically induced seizures
GABA-related drugs: pentylenetetrazol (PTZ), bicuculline and picrotoxin models These GABA_A_ receptor antagonists can serve as *models for acute seizures*. Low doses induce absence seizures while moderate doses induce clonic seizures and high doses cause tonic–clonic seizures. These seizure episodes generally resolve in relatively short time. However, continuously high doses may lead to generalized status epilepticus and eventually death.
Glycine-related drugs: strychnine model This glycine receptor blocker can serve as a model of therapy-resistant seizures arising from the lower brainstem and spinal cord.
Glutamate-related drugs: kainic acid model This glutamate receptor agonist can serve as a *model for temporal lobe epilepsy*. A single large dose or repeated lower doses, injected systemically or intracerebrally, induce severe acute seizures (acute phase) with subsequent status epilepticus, which is followed by a quiescent period of usually several weeks (silent phase). This latent period is followed by the development of spontaneous recurrent seizures (chronic phase) and brain damage.
Acetylcholine-related drugs: pilocarpine model This cholinergic agonist can serve as a *model for temporal lobe epilepsy*. Large dosages, injected systemically or intracerebrally, induce severe acute seizures (acute phase) with subsequent status epilepticus which is followed by a quiescent period of usually several weeks (silent phase). This latent period is followed by the development of spontaneous recurrent seizures (chronic phase).
Chemical kindling models Subthreshold doses of convulsant drugs, such as pentylenetetrazol, are repeatedly injected and lead to generalized seizures. Chemical kindling can be used as a model for partial complex seizures with secondary generalization.
Electrically induced seizures
Electrical kindling model Repetitive, focal application of initially subconvulsive electrical stimulation can ultimately result in generalized seizures. Kindling may also lead to the appearance of spontaneous seizures and can be used as a *model for temporal lobe epilepsy*. Stimulation can be performed in the amygdala, hippocampus, piriform cortex, perirhinal cortex or frontal cortex and all show different features.
6 Hz corneal stimulation model In this electroshock model, a 6-Hz electrical stimulus is administered to the cornea for a prolonged period (typically 3 seconds). Corneal stimulation presents a *model for partial seizures*.
Hyperthermia induced seizures
Febrile seizure model In this model, seizures are evoked by hyperthermia, via mechanisms comparable to those of fever. The body temperature is increased in immature animals at an age when brain development corresponds to that of human infants when they are most susceptible to febrile seizures. The seizures are limbic in semiology and involve the hippocampal formation. About one third of the animals develop temporal lobe epilepsy later in life.
Sensory-evoked seizures (in genetic animal models)
Audiogenic model In the audiogenic seizure model, animals display generalized clonic or tonic–clonic seizure activity in response to intense sound stimulation. A few strains of rats [the Wistar audiogenic rat (WAR)] and mice [dilute brown non-Agouti (DBA)] are genetically susceptible to audiogenic seizures.

Currently, only one neuropeptide is being used as a therapy for epilepsy. Adrenocorticotropic hormone (ACTH) is the most commonly used treatment for infantile spasms [4]. Thyrotropin-releasing hormone (TRH) also shows promising results in clinical studies for the treatment of infantile spasms [5–7]. For other neuropeptides, data are restricted to preclinical evidence. In this review, we discuss 21 different neuropeptides that have been linked to seizure disorders. We describe how seizures influence neuropeptide levels and how certain neuropeptides can modulate seizures (summarized in Table 2). This way, we aim to give some perspectives for the development of new AEDs.

**Table 2:** Effect of neuropeptides on seizures

Peptide	Sequence (*Homo sapiens*)	Receptor/target	Expression/release in seizure models and patients	Effect on seizures	References
Adrenocorticotropic hormone ACTH	S(ac)YSMEHFRWGKPVGKKRRPVKVYPNGAEDES(phos)AEAFPLEF	MCR 1-5	Patients: – ACTH ↓ in CSF of children with infantile spasms	Patients: – Anticonvulsant that is used to treat infantile spasms	[4, 148–150]
Angiotensins Ang I Ang II (1-8) Ang III (2-8) Ang IV (3-8) Ang 1-7	DRVYIHPFHL DRVYIHPF RVYIHPF VYIHPF DRVYIHP	None AT1 and AT2 AT1 and AT2 IRAP inhibition Mas	Patients: – AT1 and AT2 ↑ in cortex and hippocampus of TLE patients Animal models: – AT1 ↑ in brain of audiogenic model – Ang II ↑ and AT1 ↑ in chronic phase, AT2↑ in acute and silent phase and Mas↑ in silent phase in hippocampus of pilocarpine model	Animal models: – Ang II, Ang III and Ang IV anticonvulsive effect in PTZ kindled mice, bicuculline and picrotoxin models – Ang IV inhibits pilocarpine-induced convulsions	[93, 94, 96–102, 104]
Arginine-vasopressine peptide AVP	CYFQNCPRG-NH_2_	V1a, V1b, V2, OTR	Animal models: – AVP ↑ in blood and hypothalamus in FS model – AVP mRNA ↑ in hypothalamus after kainic acid-induced seizures	Animal models: – Proconvulsive in FS and pilocarpine models	[74, 178–185]
Cholecystokinin CCK-8	DY(sulph)MGWMDF-NH_2_	CCK1, CCK2	Patients: – CCK ↓ in temporal cortex in TLE patients Animal models: – CCK ↓ in hippocampus in TLE models – CCK ↑ in spiny neurons and glutamatergic terminals of hippocampus in pilocarpine model	Animal models: – Anticonvulsive in PTZ and picrotoxin models	[108–113]
Corticotropin-releasing hormone CRH	SEEPPISLDLTFHLLREVLEMARAEQLAQQAHSNRKLMEII-NH_2_	CRH-R1, CRH-R2	Patients: – CRH ↑ by stress in several brain regions and thus hypothesized to be ↑ in children with infantile spasms – CRH and CRH-R1 ↑ in brain of children with generalized seizures	Animal models: – Proconvulsive	[136–138, 140, 144]
Cortistatin CST-17	DRMPCRNFFWKTFSSCK	SST1-5	Animal models: – CST mRNA ↑ in hippocampus of immature rats in kainic acid model, no change in mature rats	Animal models: – Anticonvulsive in kainic acid model	[90–92]
Endorphins Leu-enkephalin Met-enkephalin β-endorphin Big dynorphin Dynorphin-A Dynorphin-B (rimorphin)	YGGFL YGGFM YGGFMTSEKSQTPLVTLFKNAIIKNAYKKGE YGGFLRRIRPKLKWDNQKRYGGFLRRQFKVVT YGGFLRRIRPKLKWDNQ YGGFLRRQFKVVT	δ-opioid receptor δ-opioid receptor μ-opioid receptor κ-opioid receptor κ-opioid receptor κ-opioid receptor	Patients: – Leu-enkephalin ↓ in CSF FS patients – Enkephalin ↑ in hippocampus of epilepsy patients –β-endorphin ↓ or = in CSF children with infantile spasms – Prodynorphin mRNA ↑ in hippocampus of TLE patients Animal models: – Proenkephalin mRNA and peptides ↑ in hippocampus in kainic acid model – Met-enkephalin ↑ in brain in FS model – Dynorphin expression in hippocampus ↓ and release ↑ in kindling and kainic acid models	Animal models: – Enkephalin proconvulsive in chemical kindling models – β-endorphin induces nonconvulsive limbic epileptiform activity and β-endorphin kindling induces generalized seizures – Dynorphin anticonvulsive in PTZ and kainic acid model	[108, 126–130, 132, 133, 148, 161–171]
Galanin	GWTLNSAGYLLGPHAVGNHRSFSDKNGLTS	GAL1-3	Animal models: – Galanin depletion in hippocampus soon after SE – Receptor expression not altered	Animal models: – Anticonvulsive in acute and chronic epilepsy models – GAL1 KO mice develop spontaneous seizures – Prevention hyperthermia-induced seizures	[38–44, 49, 50]
Ghrelin	GSS(oc)FLSPEHQRVQQRKESKKPPAKLQPR	GHSR1a	Patients: – Contradictory results; mostly ghrelin ↓, (sometimes = or ↑) in plasma epileptic patients Animal models: – Ghrelin ↓ in blood in PTZ model	Animal models: – Anticonvulsive in PTZ model – Neuroprotective for seizure-induced neurodegeneration in pilocarpine and kainic acid models	[53, 54, 58, 59, 63, 64]
Melanin-concentrating hormone MCH	DFDMLRCMLGRVYRPCWQV	MCHR1, MCHR2	Unknown	Animal models: – Acute anticonvulsive effect in PTZ model – Long-term proconvulsive effect	[199, 200]
Nesfatin-1	VPIDIDKTKVQNIHPVESAKIEPPDTGLYYDEYLKQVIDVLETDKHFREKLQKADIEEIKSGRLSKELDLVSHHVRTKLDEL	Unknown	Patients: – Nesfatin ↑ in plasma and saliva of epilepsy patients Animal models: – Nesfatin ↑ in plasma in kainic acid model	Unknown	[202–204]
Neuropeptide Y NPY	YPSKPDNPGEDAPAEDMARYYSALRHYINLITRQRY-NH_2_	Y1-Y5	Patients: – NPY and NPY mRNA ↑ in hippocampus of TLE patients – Y1 ↓ and Y2 ↑ in hippocampus of TLE patients with sclerosis Animal models: – NPY ↑ in hippocampus and cortex in several epilepsy models – NPY ↑ in hippocampus in FS model	Animal models: – Anticonvulsive in different models of acquired and genetic epilepsy	[8–11, 16–19, 21, 29, 32, 33]
Neurotensin	pQLYENKPRRPYIL	NTS1-3	Animal models: – Neurotensin ↓ in cortex and hippocampus of kainic acid model in acute phase – No alterations in the brain of kindling model	Animal models: – Anticonvulsive in the 6Hz corneal stimulation model	[118, 120, 121]
Oxytocin	CYIQNCPLG-NH_2_	OTR, V1a	Patients: – Oxytocin ↑ in plasma of TLE patient Animal models: – Oxytocin mRNA↑ in hypothalamus in kainic acid model – Oxytocin mRNA ↓ acute in hypothalamus in PTZ model – Oxytocin ↑ in plasma in PTZ model – No changes in kindling model	Animal models: – Effect dose-dependent: high doses are proconvulsive and low doses are anticonvulsive in PTZ model – OTR null mice show increased seizure susceptibility	[180, 189, 190, 192, 193, 195–197]
Pituitary adenylate cyclase-activating polypeptide PACAP	HSDGIFTDSYSRYRKQMAVKKYLAAVLGKRYKQRVKNK-NH_2_	PAC1, VPAC1, VPAC2	Animal models: – PACAP mRNA ↑ in hypothalamus in kainic acid model	Animal models: – Proconvulsive effect, most likely via stimulation of AVP secretion	[187, 188]
Somatostatin SST-14 SST-28	AGCKNFFWKTFTSC SANSNPAMAPRERKAGCKNFFWKTFTSC	SST1-5	Patients: – SST ↑ in CSF children with FS or epilepsy – SST interneurons ↓ in hilus hippocampus in TLE patients Animal models: – SST interneurons ↓ in hilus in TLE models – SST ↑ in cortex and hippocampus just before and immediately after ultra-red-induced convulsions – SST ↑ in hippocampus after kindling – SST receptors ↓ in dentate gyrus after kindling (desensitization)	Animal models: – Anticonvulsive in kainic acid, picrotoxin, pilocarpine and kindling models	[67, 74, 75, 79–86]
Tachykinins Substance P Neurokinin A Neurokinin B	RPKPQQFFGLM-NH_2_ HKTDSFVGLM-NH_2_ DMHDFFVGLM-NH_2_	NK1 NK2 NK3	Animal models: – Tachykinin peptides ↓ in cortex and hippocampus in kainic acid model immediately after seizures and ↑ after 10 days – Substance P ↑ during status epilepticus	Animal models: – Proconvulsive	[118, 173–176]
Thyrotropin-releasing hormone TRH	pQHP-NH_2_	TRH-R	Animal models: – TRH and TRH mRNA ↑ and TRH-R and TRH-R mRNA ↓ in amygdala and hippocampus after electroconvulsive and amygdala-kindled seizures	Patients: – Anticonvulsant for infantile spasms (in clinical studies) Animal models: – Anticonvulsive in amygdala-kindled and kainic acid models	[5, 154–156, 210, 217]
Vasoactive intestinal peptide VIP	HSDAVFTDNYTRLRKQMAVKKYLNSILN-NH_2_	VPAC1, VPAC2	Patients: – VIP receptors ↑ in hippocampi TLE patients – VIP ↑ in serum and CSF children with seizure disorders Animal models: – Short-term ↓ VIP in PTZ and kainic acid models	Unknown	[205–208]

Abbreviations: *ac* acetylation, *ACTH* adrenocorticotropic hormone, *Ang* angiotensin, *AT* angiotensin receptor, *AVP* arginine-vasopressine peptide, *CCK* cholecystokinin, *CRH* corticotropin-releasing hormone, *CSF* cerebrospinal fluid, *CST* cortistatin, *FS* febrile seizures, *GAL* galanin receptor, *GHSR* growth hormone secretagogue receptor, *IRAP* insulin regulated aminopeptidase, *KO* knockout, *Mas* Ang 1-7 receptor, *MCH* melanin-concentrating hormone, *MCR* melanocortin receptor, *NK* neurokinin receptor, *NPY* neuropeptide Y, *NTS* neurotensin receptor, *OTR* oxytocin receptor, *PAC* PACAP receptor, *PACAP* pituitary adenylate cyclase-activating polypeptide, *phos* phosphorylation, *pQ* pyroglutamic acid, *PTZ* pentylenetetrazol, *R* receptor, *S(oc)* O-octanoyl-serine, *SE* status epilepticus, *SST* somatostatin, *sulph* sulphation, *TLE* temporal lobe epilepsy, *TRH* thyrotropin-releasing hormone, *V* vasopressin receptor, *VIP* vasoactive intestinal peptide, *VPAC* PACAP/VIP receptor, *Y* neuropeptide Y receptor

## Anticonvulsant Neuropeptides

### Neuropeptide Y

Neuropeptide Y (NPY) is one of the most studied neuropeptides in epilepsy. We here summarize the major findings and refer to other reviews for further details. NPY is abundantly expressed in GABAergic interneurons of the mammalian central nervous system (CNS), including in the hippocampus. NPY can signal through five different receptors Y1-Y5, but predominantly acts by binding to Y1, Y2, and Y5. During seizures NPY is strongly upregulated and the release of NPY is increased in the regions of the seizure, as shown in several animal models as well as in epilepsy patients [8–11]. In patients with mesial temporal lobe sclerosis, a cell-specific loss of NPY-containing interneurons in the hippocampus was observed [12]. Chronic seizure activity also alters NPY receptor expression, with an upregulation of Y2 and a downregulation of Y1 receptors [13–15].

NPY acts as an endogenous anticonvulsant, known to prevent seizures by increasing the seizure threshold. The anticonvulsant effect of NPY has been demonstrated in different models of acquired and genetic epilepsy. It is also supported by studies using transgenic animals, showing that genetically modified rats overexpressing the NPY gene are less susceptible to seizures while deletion of the NPY gene results in increased susceptibility to seizures [16, 17]. To assess the therapeutic potential of NPY, a long-lasting NPY over-expression was achieved in the rat hippocampus by local application of recombinant adeno-associated virus (AAV) vectors. This resulted in reduced generalization of seizures, delayed kindling epileptogenesis, and strong reduction of chronic spontaneous seizures [3, 18, 19].

NPY enhances inhibitory neurotransmission, and dampens excitatory neurotransmission [20]. Studies investigating which NPY receptor subtypes are most important in mediating its anticonvulsant actions give conflicting results [21]. Y2 is mainly expressed presynaptically, where NPY inhibits the presynaptic release of the excitatory transmitter glutamate and can thereby reduce hyperexcitability [22]. Also Y5 receptor activation has been implicated in decreasing glutamate release in the hippocampus and in reducing seizures [23]. In the hippocampus, NPY does not seem to affect GABAergic neurotransmission onto pyramidal neurons. In the neocortex, however, NPY can induce a long-lasting increase of GABAergic neurotransmission on pyramidal neurons. This could be mediated through the predominantly postsynaptically located Y1 receptors and would also decrease excitability in cortical circuits and thereby contribute to the powerful anticonvulsant effect of NPY in the neocortex [20, 24]. Yet, the role of Y1 is ambiguous as Y1 receptors have been reported to mediate pro- as well as anticonvulsant effects. Administration of anticonvulsant doses of NPY also results in an increase of hippocampal dopamine, via activation of sigma 1 receptors [25]. This could contribute to the anticonvulsant effect of NPY. Dopamine can both promote or inhibit hippocampal excitability and seizure activity depending on the receptor, respectively D1 or D2, that is activated, however, the net effect of increased hippocampal dopamine is an attenuation of limbic seizures via D2 receptor stimulation [26]. For more details on the mechanisms and receptors involved in the anticonvulsant actions of NPY we refer to other reviews [2, 27, 28].

NPY may also play a role in the mechanisms by which experimental febrile seizures (FS) protect the hippocampus against the occurrence of additional seizures [29]. FS are elicited by a high body temperature and affect 2-3 % of the children between 3 months and 5 years. Short or simple FS, also referred to as typical FS, are considered benign. However, complex FS, *i.e.* seizures that persist for more than 15 minutes or recurrent seizures within the same febrile illness, considerably increase the risk for the development of temporal lobe epilepsy (TLE) later in life [30, 31]. In 80–90 % of human FS, only a single seizure occurs within a febrile episode. A single hyperthermic seizure leads to a significant elevation of temperatures required to elicit a second (or third) seizure. After an experimental FS, NPY expression is increased in the dentate gyrus, CA1 and CA3 region of the hippocampus. This increase in NPY expression might be important for the protection against additional seizures as the protective effect is abolished by a Y2 antagonist. Hence, if recurrent seizures do occur within a febrile episode, they may indicate that the underlying anti-excitatory processes mediated by NPY are not normal. One study shows that children with complex FS have significantly lower plasma NPY levels than children with typical FS or controls, suggesting that patients with inadequate NPY inhibitory activity are more susceptible to complex FS [32]. Another study, however, shows no correlation between plasma NPY levels and FS [33].

The upregulation of NPY and NPY receptor expression in the dentate gyrus after seizures may also be important in seizure-induced neurogenesis, as mice lacking NPY or the Y1 receptor show a reduced basal and seizure-induced proliferation in the dentate gyrus [34]. Seizures increase adult neurogenesis in the subventricular and subgranular zones, however, the role of this increased neurogenesis in seizure generation and epileptogenesis remains elusive [35].

In summary, changes in the NPY system induced by seizures may represent an endogenous adaptive mechanism aimed at counteracting hyperexcitability underlying epileptic activity.

### Galanin

The neuropeptide galanin and its cognate receptors (GAL 1-3) are widely distributed in the mammalian CNS. Galanin is a potent endogenous anticonvulsive peptide. Several reviews discuss the role of galanin and galanin receptors in epilepsy [3, 36, 37]. We will here summarize some important findings. Seizures lead to dramatic changes in galanin expression. Status epilepticus leads to the profound depletion of galanin from the hippocampus, which may contribute to the maintenance of seizure activity [38].

Galanin receptor agonists exhibit anticonvulsant effects after intrahippocampal administration in both acute and chronic animal models of epilepsy [38, 39]. A functional disruption of the galanin gene leads to a higher seizure susceptibility. Galanin KO mice show increased propensity to develop status epilepticus after perforant path stimulation or systemic kainic acid, as well as greater severity of pentylenetetrazol (PTZ)-induced convulsions [40]. Overexpression of the galanin gene in brainstem and entorhinal cortex hinders seizure induction in all three models [40]. Galanin also seems to be effective in preventing hyperthermia-induced seizures [41].

Galanin can act on three receptors GAL 1-3, but exerts anticonvulsant effects through the receptors GAL1 and GAL2, which are both expressed in the hippocampus, but with distinct downstream signaling cascades. While GAL1, expressed in CA1, is involved in the initiation phase, GAL2, abundant in the dentate gyrus, is implicated in the maintenance phase and the severity of epileptic seizures. GAL1 receptor KO mice develop spontaneous seizures [42, 43]. In the dentate gyrus, galanin, acting through GAL2, inhibits seizures, promotes viability of hilar interneurons and stimulates seizures-induced neurogenesis [44]. Seizure activity does however not significantly alter the expression of hippocampal galanin receptors. In the hippocampus, galanin most likely exerts its effect via presynaptic inhibition of excitatory glutamatergic neurotransmission. Galanin inhibits depolarization-induced glutamate release from rat hippocampal slices [45]. Hippocampal slices from galanin KO mice release significantly more glutamate than slices from wild type mice, whereas slices from galanin overexpression mice do not show glutamate release to the same depolarization challenge [40]. Activation of the galanin receptor might lead to hyperpolarization of the presynaptic cells by opening ATP-dependent K^+^-channels and closing voltage-dependent Ca^2+^-channels and hence impeding glutamate release and the progression of seizures [46, 47]. Galanin is also capable of modulating seizure activity through interaction with serotonin. The major effect of serotonin in the hippocampus is anticonvulsant. Galanin receptors are present on serotonergic neurons in the dorsal raphe, which project to the hippocampus. Activation of the GAL1 receptor in the dorsal raphe inhibits serotonin release in the hippocampus, which increases the severity of limbic seizures. On the other hand, activation of GAL2 receptors in the dorsal raphe increases serotonin release, which translates in the inhibition of limbic seizures [48]. Since GAL1 is far more abundant in the dorsal raphe than GAL2, the net effect of galanin in the dorsal raphe is most likely GAL1 dependent. The different effects of GAL1 and GAL2 in the dorsal raphe might have important implications for the application of GAL receptor subtype specific agonists. The beneficial effect of GAL1 activation in the hippocampus might be mitigated by an undesirable inhibition of serotonergic innervations. GAL2 activation, however, has anticonvulsant effects both in the hippocampus and in the dorsal raphe.

Galanin receptors are considered as an interesting target for anticonvulsive therapy. Several synthetic agonists of galanin receptors with optimized bioavailability were shown to inhibit experimental seizures upon systemic administration in different animal models (reviewed by [36]). Galanin is also considered as an important target for the future progression of antiepileptic gene therapy. AAV vector-mediated expression and constitutive secretion of galanin has proven to significantly attenuate limbic seizure activity and prevent seizure-induced cell death in animal models [49, 50].

### Ghrelin

Ghrelin exists in two forms: desacylated ghrelin (DAG) and acylated ghrelin (AG, also referred to as ghrelin). After secretion, ghrelin is rapidly desacylated so that DAG is the main circulating peptide. Ghrelin was long supposed to be the only biologically active form, as acylation is essential for binding to the growth hormone secretagogue receptor type 1a (GHSR1a). Recently, it has been shown that DAG also has biological actions. In the CNS, the main site of ghrelin synthesis is the hypothalamus [51]. The ghrelin receptor GHSR1a is widely expressed; both peripherally and centrally, including in seizure-prone regions such as the hippocampus [52]. Ghrelin may play a role in epilepsy, but some of the current data are conflicting. For an extensive review, we refer to [53]. In a PTZ-animal model, ghrelin blood levels significantly decrease after seizures [54]. It is possible that this reduction is due to somatostatin, released during PTZ-induced seizures, as somatostatin is known to reduce the systemic plasma concentration of ghrelin. In addition, somatostatin decreases ghrelin O-acyl transferase expression, an enzyme that catalyzes the acylation of ghrelin [55]. Ghrelin reduction might also be due to the release of leptin during PTZ-induced seizures, since ghrelin is negatively regulated by leptin [56, 57]. Clinical studies on the relationship between ghrelin and epilepsy are contradictory, as some of them show increased ghrelin levels and others no changes or even a decrease. As summarized in the review of Portelli et al. [53], too many variables are present among these studies; however, the general consensus is that ghrelin levels have the tendency to decrease following epileptic episodes.

Animal models have demonstrated that ghrelin has an anticonvulsant action. Exogenous ghrelin inhibits the development and severity of PTZ-induced seizures in rats [58]. Another study shows that ghrelin is unable to prevent seizures induced by kainic acid or pilocarpine [59]. However, DAG is able to prevent status epilepticus in 60 % of pilocarpine-treated rats and significantly delay the onset of status epilepticus in kainic acid-treated rats [59]. Given the limited number of animals used in these studies, further research is needed to determine whether ghrelin is protective against seizures and against which type of epileptic seizures.

Studying the role of the ghrelin receptor GHSR1a in animal models of epilepsy gave some interesting results. Inactivation of GHSR1a by deletion, inverse agonism, or desensitization led to the attenuation of limbic seizures [60]. This indicates that both agonists and inverse agonists for GHSR1a are capable of exerting anticonvulsant effects. The GHSR1a signals with approximately 50 % maximal activity in the absence of its peptide ligands, rendering it one of the few highly constitutively active G-protein coupled receptors currently known. Ghrelin requires GHSR1a for its anticonvulsant effect, and it is desensitization and not activation of GHSR1a that is necessary to attenuate limbic seizures in vivo [60]. It has been demonstrated that ghrelin neurons have excitatory synaptic junctions on the axons of NPY neurons in the arcuate nucleus of the hypothalamus. Ghrelin increases the firing of NPY/agouti-related peptide (AgRP) neurons, which is postulated to increase the rate of secretion of GABA and maybe also NPY and AgRP [61]. Both NPY and GABA are known to have inhibitory effects on seizures. Impairment of GABA-mediated inhibitory circuits has been implicated in different forms of epilepsy in experimental animal models and in human studies. However, further research is needed as these ghrelin-NPY/GABA interactions were described only in the hypothalamic circuitry and not in cortical or limbic brain regions, which are mainly involved in the generation of seizures. In fact, in the hippocampus ghrelin did not alter extracellular GABA levels when administered intrahippocampally, indicating that the anticonvulsant action of ghrelin against limbic seizures does not involve GABA alterations in the hippocampus [60].

Ghrelin also plays a neuroprotective role and stimulates proliferation and differentiation of adult hippocampal neuronal progenitor cells [62]. Therefore, it might play a role in reducing neuronal cell loss after hippocampal damage induced by epilepsy. In this respect, it has been shown that ghrelin significantly reduces neuronal death by pilocarpine- and kainic acid-induced seizures [63, 64]. Ghrelin most likely exerts this neuroprotective effect by promoting the PI3K/Akt signaling pathway and inhibiting the mitochondrial apoptosis pathway. This appears to be mediated through the activation of GHSR1a, as a specific antagonist of GHSR1a blocks the protective effect [65].

### Somatostatin

Somatostatin has been implicated as playing a prominent role in epilepsy (reviewed by [66, 67]). Somatostatin exists in two active forms, i.e., somatostatin-14, having 14 amino acids and somatostatin-28, having 28 amino acids. Both are produced by alternative cleavage of a single proprotein. Somatostatin-14 is dominant in the brain. It is primarily known as the hypothalamic-inhibiting hormone that regulates the release of growth hormone from the anterior pituitary. Somatostatin is, however, widely distributed throughout the CNS. In most brain regions, its function remains unclear. Somatostatin can act on five different receptors, SST-1–5. SST-1–4 are expressed in the hippocampus, and SST-2–4 likely participate in the anticonvulsant action of the somatostatin peptide. SST-1 acts as an inhibitory autoreceptor and is most likely not involved in the anticonvulsant effects of somatostatin [68]. In the hippocampus, the somatostatin peptide is mostly expressed in GABAergic interneurons in the hilus. These somatostatin interneurons represent about 16 % of the total GABAergic neurons in the dentate region. They also express mGluR1 and substance P receptors and about 30 % coexpress NPY. These interneurons project to the outer molecular layer onto dendrites of dentate granule cells, adjacent to perforant path synapses, and inhibit these granule cells. Somatostatin receptors are mainly located on granule cells. Hence, these somatostatin interneurons could modulate excitatory input that comes from the entorhinal cortex via the perforant path into the hippocampus. Their dendrites remain in the hilus, where they receive input from mossy fiber collaterals of dentate granule cells. This circuitry provides inhibitory feedback control on dentate granule cells [67]. After repetitively induced seizures, the density of somatostatin interneurons in the hilus of the dentate gyrus significantly decreases. This is shown in several animal models of TLE and is confirmed in TLE patients [12, 69, 70]. In fact, the loss of somatostatin interneurons is considered as a hallmark of epileptic hippocampus. It is still unclear, however, why somatostatin interneurons are so vulnerable to seizures-induced death and how exactly this neuronal loss contributes to epileptogenesis or post-seizure hyperexcitability. According to one hypothesis, the loss of hilar interneurons leads to reduced GABAergic inhibition of dentate granule cells, which contributes to their hyperexcitability. This was shown after electrical stimulation-induced, self-sustaining status epilepticus (SSSE) in rats [71]. In contrast, other studies report that the loss of interneurons is not accompanied by an equivalent decrease in inhibition. Compensatory functional changes are expected to increase the inhibitory output of surviving somatostatin interneurons and in part compensate for interneuronal loss in the epileptogenic hippocampus [72]. Surviving hilar somatostatin interneurons enlarge, extend dendrites, receive increased excitatory input on distal dendrites, sprout axon collaterals in the molecular layer, and form new synapses with granule cells [73]. This way the surviving hilar interneurons inhibit more granule cells and compensate for the loss of vulnerable interneurons. Hilar neurons containing NPY are also susceptible to excitotoxic damage in some epileptic models, but their loss is less pronounced than the larger group of somatostatin-containing neurons. As NPY and somatostatin are colocalized in some hilar neurons, these might reflect the same neuronal population. Prior to the death of somatostatin interneurons, somatostatin expression is increased by seizures. The somatostatin gene contains a prototypical cAMP regulatory element site that confers activity dependence to the gene, meaning that it is turned on when neuronal activity is high. This explains the high somatostatin messenger RNA (mRNA) and peptide levels seen in seizure models. Somatostatin expression is increased in the hippocampus and cortex just before and immediately after ultra-red-induced convulsions in rat [74]. Also after hippocampal kindling, mRNA expression and peptide levels are increased in the hippocampus [75], and after kainic acid-induced seizures, ectopic expression of somatostatin in dentate granule cells and pyramidal cells was demonstrated [76]. Somatostatin also shows activity-dependent release during seizures. In an in vivo hippocampal microdialysis study and in ex vivo hippocampal slices, an increase in both basal and K^+^-stimulated release was reported after kindling [77, 78]. Children with FS also have higher somatostatin titers in CSF compared to age-matched controls [79]. The somatostatin level is highest the first hours after the seizure. In addition, children with epilepsy show significantly higher somatostatin levels in CSF. This points to an increased somatostatin release from overactive neurons and/or leakage of somatostatin from damaged or anoxic neurons. The increased release of somatostatin following seizures is likely protective. Somatostatin receptors SST-2–4 in the dentate gyrus are mainly located on granule cells, which are largely spared from seizure-induced neuronal death. However, after kindling, somatostatin receptors appear to be downregulated probably by desensitization due to the high somatostatin levels [80].

Exogenous somatostatin and agonists of the SST-2 receptor reduce the severity, total number, and duration of seizures in rats, while somatostatin antiserum has proepileptic effects [81–84]. Studying the receptors that mediate the anticonvulsant actions of somatostatin, species-specific differences were found. In rats, the anticonvulsant effects seem to be mostly mediated by SST-2. Somatostatin as well as SST-2 agonists can prevent or attenuate pilocarpine-induced status epilepticus. At the Schaffer collateral-CA1 synapses, somatostatin suppresses presynaptic glutamate release, and this effect is mediated by SST-2 [85, 86]. SST-3 and SST-4 also show anticonvulsive properties in the rat hippocampus. However, SST-2 antagonism prevents the SST-3 or SST-4 mediated anticonvulsant effects, suggesting a functional crosstalk between these receptors [87]. In mice, SST-4 mediates the anticonvulsant effects, while SST-2 does not seem to play a role in the modulation of seizure susceptibility [88, 89].

### Cortistatin

Cortistatin shares strong sequence similarity with somatostatin (11 out of 14 residues for rat and mouse, while the human peptide is extended by three residues), but is derived from a different gene. It binds to the same receptors, SST-1–5, however with lower affinity than somatostatin itself. In the cortex, cortistatin and somatostatin are expressed in partially overlapping populations of interneurons, while within the hippocampus, they seem to be predominantly colocalized in inhibitory GABAergic interneurons. Cortistatin and somatostatin are most likely regulated by different stimuli, as their precursor genes contain binding motifs for different transcriptional regulatory factors, and, unlike somatostatin, cortistatin is not significantly upregulated by increases in neural activity induced by kainic acid in adult rats [90]. In immature rats, however, cortistatin is highly expressed in the hippocampus and expression further increases after kainic acid-induced seizures [91].

Cortistatin exhibits an anticonvulsive effect against kainic acid-induced seizures in rats. Furthermore, cortistatin shows a neuroprotective role, as it markedly reduces the kainic acid-induced cell loss of cortical and hippocampal neurons [92]. This is in line with its (increased) expression in the immature brain, which shows a relative resistance to seizure-induced neuronal injury.

### Angiotensins

Besides their crucial role in cardiovascular regulation, angiotensin (Ang) peptides have been implicated in the regulation of seizure susceptibility. Five peptides, Ang I–II–III–IV and Ang 1–7, are generated from the precursor protein angiotensinogen by consecutive enzymatic steps. Ang I has little or no known biological activity. Ang II and III act through two receptors, AT1 and AT2. Ang 1–7 mainly acts via the Mas receptor and is thought to counterbalance most of the Ang II effects. Ang IV exerts its effects through inhibition of insulin-regulated aminopeptidase (IRAP), also referred to as AT4. Patients with TLE show an increased expression of the AT1 and AT2 receptors in the cortex and hippocampus [93]. In an audiogenic animal model of epilepsy, besides AT1, also angiotensin-converting enzyme (ACE) is upregulated in the brain following repetitive seizures [94]. ACE is responsible for the conversion of Ang I into Ang II. In genetic audiogenic models of epilepsy, Wistar audiogenic rats or DBA/2 mice treated with an AT1 blocker or ACE inhibitors show a significant decrease in seizure severity [94, 95]. In contrast, in a pilocarpine model, ACE expression was reduced in all phases [96]. Here, it was proposed that angiotensinogen may be directly cleaved to Ang II by the enzyme tonin, which showed increased expression in all phases. In the same pilocarpine model, an increased concentration of Ang II and the AT1 receptor was seen in the hippocampus of rats in the chronic phase, while Ang II levels and AT1 expression were very low in the acute and silent phase. The conversion of Ang II to Ang 1–7 decreased during all phases. AT2 receptor expression, on the other hand, was increased in the hippocampus in the acute and silent phase and showed no alteration in the chronic phase [96].

The peptides Ang II, Ang III, and Ang IV show anticonvulsive effects in several animal models. Intracerebroventricular administration of Ang II increases the threshold of PTZ-, bicuculline-, and picrotoxin-induced seizures in mice and decreases the intensity of seizures induced by systemic administration of PTZ. Furthermore, Ang II, Ang III, and Ang IV protect against clonic convulsions in PTZ kindled mice [97–102]. The effect of Ang II and III is most likely mediated by the AT1 receptor. It was previously reported that AT1 receptors, located pre- and postsynaptically, are capable of influencing both inhibitory and excitatory neurotransmission [103]. The anticonvulsive effect of Ang IV may be mediated through inhibition of IRAP. Ang IV is a potent and competitive IRAP inhibitor. The involvement of IRAP in seizure generation is stressed by the fact that IRAP knockout mice are less sensitive to the development of PTZ-induced seizures [104]. IRAP is known to cleave a number of bioactive neuropeptides, such as oxytocin, arginine-vasopressine peptide, substance P, neurokinin A, met-enkephalin, dynorphin A, cholecystokinin-8, and somatostatin-14, all peptides which have been implicated in seizure generation. IRAP can alter the level of these circulating peptides and hence influence seizure development. In this respect, it was shown that the ability of Ang IV to inhibit pilocarpine-induced convulsions is dependent on SST-2 receptor activation and thus is possibly mediated via the inhibition of IRAP resulting in an elevated concentration of somatostatin-14 in the brain [105]. Ang IV in addition shows an antiepileptogenic effect as it is also able to block the development of an epileptic-like state in mice.

The effect of Ang peptides on seizure susceptibility could be regulated through their close interaction with different neurotransmitter/neuromodulator systems such as dopamine, GABA, and adenosine (reviewed in detail by [99]). Anatomical, biochemical, and behavioral data point out that dopaminergic pathways play a role in Ang II-related effects. It was shown that Ang II increases the threshold of PTZ-induced seizures through a dopamine-related mechanism [106]. On the other hand, Ang II also potentiates the inhibitory action of GABA and GABAergic agonists on the PTZ seizure threshold and seizure intensity [99]. The anticonvulsant effect of Ang III and Ang IV and the antiepileptogenic effect of Ang IV in the kindling model may be (partly) mediated by adenosine-dependent mechanisms through activation of the adenosine A1 receptor [97, 107]. Adenosine inhibits synaptic activity and neurotransmitter release and is a potent anticonvulsant in different seizure models.

### Cholecystokinin

Cholecystokinin (CCK) was originally discovered in the gastrointestinal tract, but is also an abundant and widely distributed neuropeptide in the brain, with high levels in the hippocampus. Different molecular forms ranging in size from 4 to 83 amino acids have been identified (e.g., CCK-58, CCK-33, and CCK-8), all derived from the same precursor preprocholecystokinin. The sulfated octapeptide CCK-8 is the predominant form in the brain. CCK has two different receptors, CCK1 and CCK2. CCK2 is the dominant form in the brain. CCK levels are significantly altered upon seizures. In several models of TLE, hippocampal CCK immunoreactivity is reduced, either indicating decreased expression, as in the mossy fiber pathway, or potentially degeneration, as in mossy cell and basket cell terminals [108, 109]. In a mouse pilocarpine model of recurrent seizures, an increase in CCK expression was shown in spiny neurons and glutamatergic terminals, possibly originating from pyramidal cells that innervate strata radiatum and oriens of CA1, while in other layers of the hippocampus CCK expression was decreased [110]. Also in TLE patients, CCK levels were 20 % lower in the temporal cortex from which active epileptic spiking was recorded in comparison to tissue samples, which were electrographically free of epileptiform spikes [111].

CCK-8 has been demonstrated to have anticonvulsive properties in animal models by delaying the seizure onset and increasing the seizure threshold of convulsants such as PTZ and picrotoxin [112, 113].

In normal conditions, CCK is postulated to be released under strong, repetitive stimulation, activating predominantly CCK2 receptors in the brain. There is evidence that activation of CCK2 receptors in hippocampal interneurons and pyramidal cells produces a depolarizing response by decreasing potassium conductance. Accordingly, CCK can enhance GABA as well as glutamate release in the hippocampus [114, 115]. The subsequent effect depends on the cell types and precise synapses involved. In mossy fiber axons, CCK is codistributed with the opioid peptides enkephalin and dynorphin. Recurrent seizure activity increases enkephalin immunoreactivity and decreases CCK and dynorphin levels. As enkephalin can induce limbic seizures when injected into the hippocampus, while CCK has anticonvulsive properties, one might speculate that the shift in CCK/enkephalin balance in the mossy fiber system following seizures might underlie future seizure susceptibility [108, 116, 117].

### Neurotensin

Neurotensin is widely distributed in the CNS, with the highest concentrations in the limbic system. There is considerable evidence that this neuropeptide might act as a regulator of neuronal excitability. There are three known neurotensin receptors. NTS1 and NTS2 are G-protein-coupled receptors, while NTS3 is composed of a single transmembrane domain. NTS1 and NTS2 are present on neurons in the basolateral amygdala and hippocampus, which are known to be involved in seizure activity. Neurotensin-like immunoreactivity is reduced in the frontal cortex and hippocampus of rats during and after limbic seizures induced by systemic injection of kainic acid [118]. These early decreases in peptide levels may result from increased release and subsequent enzymatic inactivation of the peptides during acute seizures. Neurotensin-like immunoreactivity normalizes in the hippocampus after the period of acute convulsions, but remains decreased in the frontal cortex up to 30 days. This late decrease in peptide levels could reflect degeneration of neurotensin-containing neurons projecting from limbic brain areas to the cortex. Meanwhile, neurotensin levels increase in the striatum and substantia nigra, which might be a consequence of strong activation of the dopamine system in these areas, where neurotensin and dopamine show a close anatomical and functional relation [119]. Changes in neurotensin levels seem to be seizure-type specific, as no changes in peptide levels were shown in different brain regions in kindling models [120]. This might be explained by the fact that, in contrast to the kindling model, the kainic acid model simulates status epilepticus that is associated with hippocampal cell death.

Neurotensin and some glycosylated analogues exhibit (sub)picomolar anticonvulsant potencies in a corneal stimulation mouse model of epilepsy [121]. It is not known how neurotensin receptors mediate the anticonvulsive properties [122]. It might be related to the fact that neurotensin potentiates GABAergic activity in the rat hippocampus CA1 region [123]. Neurotensin analogues combining lipidization and cationization show improved metabolic stability and CNS bioactivity, resulting in enhanced antiseizure activity in the corneal stimulation model following intraperitoneal administration of the analogues [124].

### Dynorphins

Dynorphins belong to the family of endorphins and act through binding the opioid receptors (δ, μ, and κ). Most of dynorphin’s effects are mediated by the κ-receptor. Dynorphins are produced in different parts of the brain, including the hypothalamus, hippocampus, midbrain, medulla, pons, and spinal cord and have different physiological effects, depending on their site of production. They can modulate pain responses and maintain homeostasis through appetite control and regulation of body temperature [125]. In amygdaloid kindling and kainic acid models, dynorphin release is increased, while expression in the hippocampus is reduced, possibly as a consequence of depletion during the seizures [117, 126–128]. Also after induction of status epilepticus, a decrease in dynorphin-like immunoreactivity was observed in the dentate gyrus and CA3 of the hippocampus [129]. In TLE patients, however, a dynamic upregulation of prodynorphin mRNA expression was seen in the dentate gyrus. Highest transcript levels were seen postictally [130]. It was also shown that opioid receptor binding increases in patients within the first hours after a spontaneous seizure, suggesting an important role of the opioid system in seizure control [131].

Κ-opioid receptor agonists can prevent both behavioral and electroencephalographic measured seizures in different models of epilepsy [132] and counteract the initiation and maintenance of status epilepticus [129]. Prodynorphin knockout mice display a significantly reduced seizure threshold, faster seizure onset and prolonged seizure activity in PTZ and kainic acid models. This phenotype can be rescued by a κ-receptor-specific agonist [133]. Dynorphin peptides primarily act at presynaptic κ-opioid receptors to inhibit excitatory amino acid release from perforant path and mossy fiber terminals and can thereby reduce excitability [134].

## Anticonvulsant Neuropeptides as Treatment for Infantile Spasms

Infantile spasms are a specific type of seizures seen in an epilepsy syndrome of infancy and childhood known as West syndrome. The term infantile spasm is also used as a synonym for this syndrome. The underlying causes may include perinatal insults, structural abnormalities, neurocutaneous syndromes, metabolic diseases, CNS infections, or genetic disorders [135].

### Adrenocorticotropic Hormone

ACTH, also known as corticotropin, is together with β-endorphin derived from the proopiomelanocortin (POMC) precursor. ACTH acts through binding the melanocortin receptors (MCR). Five receptors (MCR 1–5) have been identified, however, whether ACTH is an endogenous ligand of all these receptors has not been determined. Today, ACTH is the most commonly used first-line treatment for infantile spasms due to an unknown etiology or due to structural/metabolic etiology [4].

The stress/corticotropin-releasing hormone (CRH) hypothesis proposes that all the etiologies of infantile spasms share the common characteristic of being stressful to the immature brain causing an increase in the release of stress-activated mediators, especially the neuropeptide CRH in limbic, seizure-prone brain regions [136, 137]. Animal studies confirm that intracerebroventricular infusion of CRH results in seizures in young rats. These CRH-induced seizures originate from the amygdala and then spread to the hippocampus [138]. CRH can act through binding two receptors, CRH-R1 and CRH-R2, which are both present in the amygdala. CRH-R1 is most likely responsible for mediating the convulsant effects of CRH in the developing brain, as selective CRH-R1 antagonists increase the latency and decrease the duration of CRH-induced seizures [139]. In addition, elevated CRH and CRH-R1 expression was found in postmortem brain from children with generalized seizures, while no significant difference in the expression of CRH-R2 was observed [140]. Activation of CRH receptors in the amygdala and hippocampus might lead to hyperexcitability by suppression of afterhyperpolarisation and potentiation of glutamatergic neurotransmission [141, 142]. Hence, excess secretion of CRH might trigger seizures. A drastic decline of CRH receptors, occurring with maturation, would be responsible for the reduction in this peptide-mediated hyperexcitability later in life [143]. Animal studies confirm that CRH-induced seizures are considerably less prominent in adult rats compared to infant rats [144].

ACTH and oral steroids can ameliorate infantile spasms by reducing the synthesis and release of endogenous CRH [145, 146]. ACTH is produced and secreted by the anterior pituitary in response to hypothalamic CRH and promotes the release of adrenal steroids (primarily cortisol) into the bloodstream. The effect of ACTH on steroid secretion is mediated by MCR-2. Steroid hormones have a multitude of effects throughout the body, among which suppression of the proconvulsant neuropeptide CRH. ACTH can also act on melanocortin receptors (most likely MCR-4) located on CRH-expressing neurons in the amygdala and, hence, suppress CRH expression independently of adrenal steroids [147]. This explains the enhanced potency of ACTH compared to steroids in the treatment of infantile spasms. Children with infantile spasms show reduced ACTH levels in CSF [148–150]. This is in line with the expected high CRH levels. Chronic activation of CRH receptors leads to their desensitization, which decreases ACTH release [151]. ACTH is currently one of the recommended therapies for infantile spasms, but its efficacy is not universal and prolonged administration can lead to serious side effects [152]. Agents who either activate melanocortin receptors or block the seizure-promoting action of CRH could present therapeutic alternatives.

### Thyrotropin-Releasing Hormone

TRH is a tripeptide that is released from the hypothalamus and stimulates the release of thyroid-stimulating hormone and prolactin from the anterior pituitary. TRH is also expressed in other brain regions including the cerebral cortex, hippocampus, amygdala, striatum, and brainstem. A single TRH receptor gene has been found in humans, while two genes encoding homologous receptors, TRH-R1 and TRH-R2, are present in rodents. TRH-R1 is the major form in the anterior pituitary gland, while TRH-R2 is present in other brain sites [153]. TRH and TRH mRNA are substantially upregulated over several days after electroconvulsive and amygdala-kindled seizures, whereas the TRH receptor and receptor mRNA are downregulated in seizure prone areas such as the amygdala and hippocampus [154–156].

Clinical studies show that TRH can be used to treat infantile spasms [5–7]. The effectiveness of TRH has been reported in West syndrome, Lennox–Gastaut syndrome, and early infantile epileptic encephalopathy that were intractable to ACTH and other conventional anticonvulsants. It was proposed that TRH may act as an antiepileptic through a kynurenine mechanism, as TRH increases CSF kynurenine levels in patients with intractable epilepsy and kynurenic acid acts as an antagonist on the NMDA receptor complex [157]. In the rat hippocampus, it was shown that TRH also increases GABA release, which may also contribute to its antiepileptic effects [158]. Further studies are required to elucidate the exact mechanism of its antiepileptic action. TRH is considered as a possible new treatment for children with infantile spasms, as side effects were minimal in the first clinical studies. The peptide itself is however a poor drug candidate due to its short plasma half-life (5 min), poor CNS permeability, and endocrine side effect. Therefore, selective TRH analogs that are metabolically stable and more potent are being developed [159].

## Proconvulsant Neuropeptides

### β-Endorphin

β-Endorphin is a neuropeptide that is derived from the same POMC precursor as ACTH. β-Endorphins are present abundantly in the hypothalamus and pituitary gland and are released when the body encounters stress or pain. During pain, they exert an analgesic effect. During stress, they are released in the limbic system and reduce the extent of anxiety. β-Endorphin exerts its effect through binding the opioid receptors, with highest affinity for the μ-receptor. μ-Receptors are located presynaptically and inhibit the release of the inhibitory neurotransmitter GABA and cause more dopamine to be released [125]. As for ACTH, the release of β-endorphin is promoted by CRH. Hence, β-endorphin levels could be influenced in patients with infantile spasms; however, current data are conflicting. In one study, β-endorphin and ACTH show decreased levels in the CSF of children with infantile spasms [148]. In contrast, another study shows normal levels of β-endorphin and reduced ACTH levels in the CSF of patients with infantile spasms and thus an increased β-endorphin/ACTH ratio [160].

β-Endorphin induces nonconvulsive limbic epileptiform activity when administered intraventricularly to rats, suggesting that β-endorphin plays a role in regulating limbic excitability [161]. Repeated microinjection of β-endorphin into the amygdala or hippocampus leads to the development of generalized convulsions, an effect that could be antagonized by a μ-receptor specific antagonist [162].

### Enkephalins

Leu- and met-enkephalin are both pentapeptides with respectively C-terminal leucine and methionine and are derived from the proenkephalin precursor. They belong to the family of endorphins and act through binding the opioid receptors, with highest selectivity for the δ-opioid receptor. Several studies report changes in enkephalin levels upon seizures (reviewed in detail by [2]). In animal models, limbic seizures induce dramatic increases in enkephalin immunoreactivity in the hippocampus, most prominently within the mossy fibers [117]. In fact, a single systemic kainate injection in adult rats upregulates proenkephalin mRNA and its peptide products in the hippocampus for at least 1 year [163]. Also in patients with generalized epilepsy, an increase in enkephalin immunoreactivity was found in the CA regions of the hippocampus [164]. This suggests an important role for this peptide in the occurrence of recurrent, unprovoked seizures in TLE. In the neonatal rat brain, elevated met-enkephalin levels were also observed immediately after hyperthermia-induced seizures [165]. Leu-enkephalin levels, however, are significantly lower in the CSF of patients with FS [166].

There is strong evidence from animal models that enkephalins act as proconvulsive agents. Met-enkephalin was shown to have an epileptogenic effect in the rat brain. Repeated microinjection of met-enkephalin into the amygdala led to the development of generalized convulsions [167]. A δ-receptor agonist also effectively kindled convulsions when microinjected into the amygdala or ventral hippocampus [162]. In addition, a δ-receptor selective agonist was shown to produce a brief, nonlethal convulsion in mice, similar to the effect of PTZ [168]. Activation of δ-receptors also increases the incidence of bicuculline-induced convulsions in mice [169]. On the other hand, δ-receptor antagonists were shown to depress PTZ-induced kindling in rats [170, 171]. Enkephalins are known to inhibit spontaneous GABA release from inhibitory neurons in the hippocampus, resulting in increased excitability [172].

### Tachykinins

Tachykinins are widely distributed throughout the central and peripheral nervous system, and numerous functions have been attributed to them. Tachykinins have also been implicated in epilepsy. In humans, two tachykinin genes have been described, TAC 1 and TAC 3. TAC 1 encodes the peptides neurokinin A, substance P and two N-terminally extended forms of neurokinin A, neuropeptide K, and neuropeptide γ. TAC 3 encodes neurokinin B. Substance P acts through the NK1 receptor and neurokinin A and B act through the NK2 and NK3 receptor, respectively. Seizures alter the concentrations of substance P, neurokinin A, and neurokinin B [118, 173]. Immunoreactivity for substance P and neurokinins significantly decreases in the frontal cortex, dorsal hippocampus, and striatum of rat brains immediately after kainic acid-induced seizures. One to 3 days after injection of kainic acid, peptide levels recover, and 10–60 days later, they are significantly increased in the frontal cortex and the hippocampus. NK1 is expressed on somatostatin-containing interneurons, which are significantly decreased in TLE patients and in several animal models after repetitive seizures [67].

Tachykinins are involved in generating limbic seizures and in hippocampal-selective neuronal vulnerability. Mice with disruption of the TAC1 gene show a reduction in duration and severity of seizures induced by kainic acid or PTZ resulting in the prevention of both necrosis and apoptosis of hippocampal neurons [174]. Systemic administration of a NK1 antagonist reduces kainic acid-induced seizure activity, supporting a proconvulsive action for substance P [175]. Other studies suggest that substance P contributes to both the initiation phase and maintenance phase of SSSE [176]. During SSSE, the expression of substance P is enhanced. Intrahippocampal injection of substance P triggers SSSE, while injection of NK1 antagonists prevented and stopped it. The substance P mediated excitation of hippocampal neurons might be explained by an increase in glutamate release [176].

### Arginine-Vasopressine Peptide

Arginine-vasopressine peptide (AVP) is synthesized in the supraoptic and paraventricular nuclei of the hypothalamus, from which AVP-containing neurons project to the posterior pituitary, amygdala, and septum. Three AVP receptors (V1a, V1b, and V2) exist in mammals, and, in addition, AVP shows nanomolar affinity for the oxytocin receptor (OTR). V1a is widely distributed in peripheral tissues and different areas of the CNS, including the cerebral cortex, hypothalamus, hippocampus, and brainstem. V1b is mostly expressed in the pituitary gland and also in other brain regions and peripheral tissues. V2 is mainly present in the kidney, but is also present in the brain, where it was demonstrated in the hippocampus of newborn rats [177]. AVP acts as an endogenous antipyretic, attenuating fever by influencing the thermoregulatory neurons in the anterior and preoptic region of the hypothalamus and in adjacent septal areas. Although the antipyretic effect of AVP release during fever is beneficial, excessively high levels of AVP may be detrimental. There is evidence that increased levels of AVP released during fever may be involved in the precipitation of FS [178]. Rats that genetically lack AVP and rats passively immunized with anti-AVP antiserum show an increased body temperature threshold for convulsions [179]. High blood levels of AVP in hyperthermic convulsing rats compared to hyperthermic nonconvulsive rats also support the hypothesis that AVP may mediate febrile convulsions. AVP levels in the hypothalamus increase in a rat model of FS immediately after ultra-red irradiation and significantly decrease after convulsions, when body temperature is normalized [74].

The role of AVP is not restricted to febrile convulsions. Also in other seizure models, there is evidence that AVP exerts proconvulsant effects. AVP mRNA levels are increased following kainic acid-induced seizures [180, 181]. Intraventricular AVP administration leads to convulsions or abnormal behaviors associated with epileptic discharges in rats [182, 183]. Subcutaneously administered AVP potentiates pilocarpine-induced seizures [184]. Studies with antagonists for the AVP receptors V1 and V2 provide further evidence that AVP has a convulsant activity mediated by V2 receptor activation. V2 receptor antagonists not only prevent hyperthermic convulsions but also the status epilepticus induced by pilocarpine in adult rats [185].

### Pituitary Adenylate Cyclase-Activating Polypeptide

Pituitary adenylate cyclase-activating polypeptide (PACAP) belongs to the glucagon–secretin–vasoactive intestinal peptide (VIP) family of peptides. PACAP is expressed in various brain regions where it serves neurotrophic, neuromodulatory, and neurotransmitting functions. High concentrations are found in the neocortex, hypothalamus, hippocampus, and brainstem. PACAP can activate three different subtypes of receptors which are widely distributed in the CNS: the PACAP-specific PAC1 receptor and two PACAP/VIP-indifferent VPAC1 and VPAC2 receptors. PACAP is one of the main regulators of the neurohypophyseal secretion of AVP [186]. Systemic administration of PACAP in rats leads to a decrease in the threshold for the development of FS [187]. This is most likely an indirect effect as PACAP stimulates AVP neurosecretion and AVP is known to mediate hyperthermia-induced seizures [179]. However, it has also been shown that PACAP mRNA is increased in the paraventricular nucleus of the hypothalamus after kainic acid-induced seizures in rats [188]. More studies are needed to investigate the role of PACAP in seizure susceptibility.

## Neuropeptides with Less Clear Overall Anti-/Proconvulsant Effects

### Oxytocin

Oxytocin is synthesized in neurons of the supraoptic and paraventricular nuclei of the hypothalamus. The oxytocin gene shares considerable homology and a common ancestral origin with the vasopressin gene. These peptides are also found in adjacent neurons within the hypothalamic nuclei, which have similar types of synaptic inputs and respond to similar physiological stimuli for release. Oxytocin binds to both the OTR and the vasopressin 1a receptor (V1a).

A case report of a prolonged temporal lobe epileptic seizure revealed that both vasopressin and oxytocin plasma concentrations are elevated during the generalized phase of a seizure [189]. Also in the rat PTZ model, oxytocin plasma levels are consistently elevated [190]. Following kainic acid-induced seizures, both oxytocin and vasopressin mRNA levels in hypothalamic nuclei are increased [180]. Another study showed that oxytocin-containing neurons of the paraventricular nucleus are activated following generalized seizures [191]. On the other hand, in the kindled-seizure model, no changes in oxytocin expression were found [192], and in the PTZ model, hypothalamic oxytocin mRNA is significantly reduced immediately after treatment [193].

Contradictory results have been described concerning the role of oxytocin in seizures. In 1978, a case report described a patient that experienced a generalized epileptic convulsion after intra-amniotic prostaglandin F2 alpha administration together with intravenous oxytocin infusion [194]. In the mouse PTZ model, it was also shown that oxytocin has proconvulsive properties and these are mediated by the V1a receptor [195]. These effects were seen after a single high dose of oxytocin (0.5 mg/kg ∼500 nmol/kg). A lower dose of 0.25 mg/kg did not alter seizure susceptibility. In contrast, lower doses of oxytocin were shown to be anticonvulsive in a rat model of PTZ-induced seizures. Respectively 40, 80, and 160 nmol/kg oxytocin were administered once daily for five consecutive days, after which EEGs were recorded. The two highest doses presented a decreased convulsion scale and less EEG abnormalities [196]. Also in zebrafish, oxytocin and its nonmammalian homologue isotocin show anticonvulsive effects leading to a decreased number of seizures after a PTZ injection [197]. This suggests that the dose of oxytocin is crucial and high doses might lead to seizures. OTR null mice have an increased susceptibility for seizures, a characteristic that was antagonized by peripherally administered oxytocin. The latter is probably explained by a possible upregulation of V1a and an interaction of oxytocin with these receptors. Because hippocampal neurons from OTR null mice express a lower percentage of GABAergic synapses, it is possible that an imbalance in glutamate–GABA transmission is responsible for the increased seizure susceptibility [198].

### Melanin-Concentrating Hormone

Melanin-concentrating hormone (MCH) is a neuropeptide produced primarily in the lateral hypothalamus and zona incerta. It interacts with two G-protein coupled receptors, the melanin concentrating hormone 1 and 2 receptor (MCHR1 and MCHR2), of which only MCHR1 is expressed in rodents. MCH neurons project to several brain regions with known involvement in epileptic seizures. MCHR1 is expressed throughout the brain with particularly dense localization in the nucleus accumbens shell, hippocampus, amygdala, locus coeruleus, and cerebral cortex.

Central MCH administration evokes a strong anticonvulsant effect in PTZ-treated rats [199]. Comparisons between MCHR1-KO and WT mice, however, show that inactivation of the MCH signaling system decreases susceptibility to several types of seizures [200]. The acute anticonvulsant effect of MCH could be explained by an immediate inhibitory effect on neuronal firing in certain brain regions, mediated by MCHR1 that couples to Gi. The long-term proconvulsant role for MCH could be a consequence of alterations in NMDA receptor expression, as MCH administration has been reported to positively regulate NMDA receptor expression in the hippocampus. This phenomenon of “effect inversion” where the effects of acute administration of an agonist or antagonist/inverse agonist are opposite to those seen with chronic administration is well documented for adenosine, which is also involved in the regulation of seizures [200].

## Other Neuropeptides that Show Potential in the Field of Epilepsy

### Nesfatin-1

Nesfatin-1 or NEFA/NUCB2-encoded satiety and fat-influencing protein is a recently discovered neuropeptide expressed in several neurons of the forebrain, hindbrain, brainstem, and spinal cord and is known as a potent anorexigenic substance, playing a role in the hypothalamic pathways regulating food intake and energy homeostasis [201]. The receptor through which nesfatin-1 exerts its actions is yet to be identified. Correlations have been found between nesfatin-1 levels in body fluids and the course of epilepsy. Patients with primary and secondary generalized seizures show significantly elevated nesfatin-1 levels in plasma and saliva [202]. The plasma nesfatin-1 level is in the highest range during 5 min after onset of the attack [203]. In addition, plasma nesfatin-1 level is significantly elevated in rats with epileptic seizures induced by kainic acid [204]. The mechanism responsible for this increase is not known. However, nesfatin-1 can be considered as a promising marker to diagnose patients who have suffered a recent epileptic seizure.

### Vasoactive Intestinal Peptide

Vasoactive intestinal peptide (VIP) belongs together with PACAP to the glucagon–secretin–VIP family of peptides. VIP can activate two receptors, VPAC1 and VPAC2, in the CNS. VIP can enhance the electrical activity of neurons in many brain regions and could therefore play a role in seizure disorders. This peptide, however, has not been heavily investigated in this respect. A short-term and transient decrease in VIP levels was reported after PTZ and kainic acid-induced seizures in rats [205, 206]. On the other hand, VIP levels were elevated in serum and CSF of children with seizure disorders [207]. A significant upregulation of VIP receptors without a change in pattern and distribution of VIP immunoreactive neurons was seen in the seizure focus of human hippocampi removed surgically from patients with medically intractable TLE [208]. An upregulation of VIP receptors on pyramidal neurons might point to an increased excitation of these cells by VIP. If VIP functions as a trophic substance, the increased VIP receptor level might also protect neurons from excitotoxic injury. Another hypothesis is that VIP stimulates glycogenolysis to cope with the increased energy demands of hippocampal seizure onset.

## Perspectives

Several endogenous peptides act as neuromodulators that can suppress seizures in the brain. Examples are ACTH, angiotensin, CCK, cortistatin, dynorphin, galanin, ghrelin, NPY, neurotensin, somatostatin, and TRH. Other neuropeptides, such as AVP, CRH, enkephalins, β-endorphin, PACAP, and tachykinins, show proconvulsive properties. These neuropeptides and their receptors open new avenues towards generating AEDs.

Neuropeptides are attractable drug targets due to their high potency, selectivity for their target receptors, and low toxicity of their metabolites. Despite the growth in understanding the role of neuropeptides in epilepsy, peptide-based therapeutics are staying behind. Several things need to be considered when using neuropeptide-related targets in the treatment of epilepsy. A major concern is the disruption of the normal physiological function(s) of the peptides. Other drawbacks of neuropeptides are their rapid enzymatic degradation and the fact that many neuropeptides cannot penetrate the blood–brain barrier (BBB). However, new technologies open opportunities for CNS focused peptide-drug delivery. Recently, significant progress has been made in generating subtype-selective, metabolically stable, CNS-penetrant analogs of various anticonvulsant neuropeptides [122]. For galanin, NPY, and neurotensin, the combination of acetylation, cationization, and lipidization was shown to produce an overall improvement on metabolic stability, generating BBB-permeable, systemically active analogs that protect against seizures [124, 209]. Improved delivery systems may further help in circumventing the BBB. Intranasal delivery of neuropeptide-loaded biodegradable nanoparticles provides a promising approach in seizure therapy. Neuropeptides are delivered directly to specific CNS targets by transport through the olfactory epithelium. As a proof of concept, it was shown that intranasal delivery of TRH/analog nanoparticles can significantly attenuate seizures in a kindling model [210, 211]. Gene therapy on candidate neuropeptide genes also shows promising anticonvulsive effects in preclinical studies [212]. AAV vectors support long-term, nontoxic gene expression in the CNS, and these AAV properties prove particularly applicable to the treatment of focal epilepsies, especially intractable TLE [213]. A hybrid AAV vector can selectively cross the seizure compromised BBB and transduce cells after peripheral, intravenous administration. Hence, AAV therapeutics for focal epilepsies may be delivered without any neurosurgical interventions [214]. Moreover, multiple genes can be expressed from the same vector, meaning that it should be possible to test the synergy of numerous combinations of anticonvulsant neuropeptides [215]. Extensive studies on the inhibitory neuropeptides galanin and NPY have generated enough preclinical evidence of efficacy to warrant AAV-based clinical trials.

As neuropeptide expression is significantly altered in specific epileptic conditions, neuropeptides might also be considered as biomarkers. Since the treatment of seizures depends on an accurate diagnosis, making sure that a person has epilepsy and knowing what kind is a critical first step. There are many other disorders that can cause changes in behavior and can be confused with epilepsy. Nesfatin-1 could, e.g., be a candidate biomarker, as nesfatin-1 levels are significantly elevated in plasma and saliva of epilepsy patients that experienced a recent attack [202, 203].

Finally, there is a pressing need for drugs that are truly antiepileptogenic, i.e., drugs that prevent the process by which the brain becomes epileptic. To evaluate the potential of endogenous neuropeptides as disease-modifying drugs more (pre)clinical studies are needed.

## Footnotes

1 Table 1 gives an overview of all animal seizure models referred to in this review.
